# Metabolomics Study Reveals Inhibition and Metabolic Dysregulation in *Staphylococcus aureus* Planktonic Cells and Biofilms Induced by Carnosol

**DOI:** 10.3389/fmicb.2020.538572

**Published:** 2020-09-18

**Authors:** Fengge Shen, Chunpo Ge, Peng Yuan

**Affiliations:** ^1^Xinxiang Key Laboratory of Molecular Neurology, School of Basic Medical Sciences, Xinxiang Medical University, Xinxiang, China; ^2^School of Public Health, Xinxiang Medical University, Xinxiang, China

**Keywords:** methicillin-resistant *Staphylococcus aureus*, carnosol, biofilm, antiadhesion, antimicrobial activity, metabolomics

## Abstract

*Staphylococcus aureus* (*S. aureus*) is a global health threat accompanied by increasing in drug resistance. To combat this challenge, there is an urgent need to find alternative antimicrobial agents against *S. aureus*. This study investigated the antimicrobial efficacy of carnosol against *S. aureus* using an *in vitro* model. The effects of carnosol were determined based on the antimicrobial effects or formation and disruption of biofilms. Finally, metabolomics of *S. aureus* grown as planktonic cells and biofilms with carnosol treatment were analyzed using gas chromatography-mass spectrometry. The minimum inhibitory concentrations (MICs) of carnosol were 32 to 256 μg/mL against the sixteen tested *S. aureus* strains. Among the biofilms, we observed a reduction in bacterial motility of the *S. aureus*, biofilm development and preformed biofilm after carnosol treatment. Moreover, the significantly altered metabolic pathways upon carnosol treatment in *S. aureus* planktonic cells and biofilms were highly associated with the perturbation of glyoxylate and dicarboxylate metabolism, glycine, serine and threonine metabolism, arginine and proline metabolism, alanine, aspartate and glutamate metabolism, arginine biosynthesis, and aminoacyl-tRNA biosynthesis. In addition, glutathione metabolism, D-glutamine and D-glutamate metabolism were significantly changed in the biofilms. This study establishes the antibacterial and antibiofilm properties of carnosol, and will provide an alternative strategy for overcoming the drug resistance of *S. aureus*.

## Introduction

*Staphylococcus aureus* (*S. aureus*) *is* one of the major gram-positive opportunistic pathogens associated with hospital-acquired infections and food-borne illnesses worldwide (Knox et al., 2015). The emergence and spread of methicillin-resistant *S. aureus* (MRSA) strains and the formation of biofilms make *S. aureus* one of the most difficult challenges to control (Moormeier and Bayles, 2017; Le et al., 2020). In February 2017, the World Health Organization (WHO) listed MRSA as a “High Priority” pathogen for the development of new antibiotics (Willyard, 2017).

In nature, most microorganisms can exist in two forms: a planktonic form or as a biofilm. Once microorganisms form a biofilm, their genetic expression changes rapidly, which makes them physiologically and metabolically distinct from their corresponding planktonic cells (Kaiser et al., 2017). Biofilms often exhibit a strong inherent resistance to antimicrobial drugs, which makes the control of biofilm infections very difficult, and this may be further troubled if the pathogenic microorganism is resistant to multiple agents.

To combat this challenge, there is an urgent need to develop novel therapeutic drugs or antibiotic alternatives that are active against *S. aureus*. One approach is to develop new antimicrobial agents, whereas another approach is combination therapy to improve the efficacy of antimicrobials for difficult infections (Nguyen and Graber, 2010). In addition, plants and other natural materials may prove to be valuable sources of useful new antimicrobials or antimicrobial synergists (Shan et al., 2007; Gossan et al., 2016; Yang et al., 2017).

Carnosol is a natural polyphenol (dietary diterpene) extracted mainly from the Lamiaceae family (mint family), consisting of plants such as sage, rosemary, lavender, and oregano (Horiuchi et al., 2007). Carnosol and carnosic acid are known to be among the most potent natural antioxidants used for food preservation but also have health benefits, such as detoxification and anti-inflammatory effects (Sanchez et al., 2015). Recently, many reports have shown that it has multiple pharmacological effects, including antifibrosis, antitumor, antifungal, anti-inflammatory, neuroprotective, and antioxidant activities, in multiple cell lines and animal models by regulating relevant molecular pathways (Johnson, 2011; De Oliveira, 2016; Seow and Lau, 2017; Valdés et al., 2017; Ramírez et al., 2018; Zhao et al., 2018; Yeo et al., 2019). Moreover, carnosol also showed activity against *S. aureus*, *Streptococcus pyogenes*, *Bacillus subtilis* and *S. epidermidis* (Encarnación-Dimayuga et al., 1998; Collins and Charles, 1987; Ferreira et al., 2016; Chabán et al., 2019). Only MICs with carnosol treatment were investigated in MRSA *and* MSSA, but the mechanism of action was underexplored. The biological effects of carnosol on *S. aureus* is not entirely clear, especially in *S. aureus* biofilm, which limits our understanding of the medicinal effects of carnosol.

In the present study, we investigated the antimicrobial efficacy of carnosol against standard and clinical strains of *S. aureus* grown in planktonic and biofilm cultures by adopting an *in vitro* model. Moreover, cellular metabolic perturbations were investigated by metabolomics in *S. aureus* planktonic cells and biofilms in response to carnosol treatment.

## Materials and Methods

### Bacterial Strains and Materials

Standard *S. aureus* strain ATCC 29213 was provided by the China Medical Culture Collection Center (Beijing, China). Ten clinical strains of *S. aureus* (MRSA 4642 and 4670; MSSA 4611 and 4672; *S. aureus* 4563, *S. aureus* 4612, *S. aureus* 4623, *S. aureus* 4643, *S. aureus* 4644 and *S. aureus* 4474) were isolated from the China-Japan Friendship Hospital. Five MRSA strains, MRSA 3183, MRSA 2961, MRSA 2047, MRSA 41122 and MRSA 3335, were isolated from the First Hospital of Jilin University, Changchun, China. This study was conducted in accordance with the declaration of Helsinki. This study was conducted with approval from the Ethics Committee of Xinxiang Medical University. Carnosol was purchased from Sigma-Aldrich, and a stock solution was dissolved in dimethyl sulfoxide (Sigma-Aldrich). Seven antibiotic drugs (penicillin G, vancomycin, oxacillin, ciprofloxacin, levofloxacin, tetracyclines and gentamicin) were purchased from Beijing Solarbio Science & Technology Co., Ltd., Beijing, China. All strains were grown in tryptic soy broth medium (TSB, Qingdao Hope Bio-Technology Co., Ltd., Qingdao, Shandong, China) at 37°C.

### Planktonic Antimicrobial Susceptibility Testing

The minimum inhibitory concentrations (MICs) of carnosol and seven antibiotic drugs, penicillin G (PEG), vancomycin (VAN), oxacillin (OXA), ciprofloxacin (CIP), levofloxacin (LVX), tetracyclines (TET) and gentamicin (GEN), against the sixteen *S. aureus* strains were determined by microdilution in cation-adjusted Mueller-Hinton broth (CA-MHB, Qingdao Hope Bio-Technology Co., Ltd., Qingdao, Shandong, China) using the Clinical and Laboratory Standards Institute (Clinical and Laboratory Standards Institute [CLSI], 2012a). Oxacillin wells were supplemented with 2% NaCl. Briefly, overnight cultures grown in CA-MHB were diluted to 10^6^ cfu/mL in CA-MHB. One hundred microliters of bacterial suspension and 100 μL of carnosol (16-512 μg/mL) or antibiotics were transferred to a 96-well microtiter plate at 35°C for 16–24 h. The MIC was defined as the lowest concentration at which no visible bacterial growth was observed. MIC breakpoints were used to define susceptible (S), intermediate (I), and resistant (R) strains (Clinical and Laboratory Standards Institute [CLSI], 2012b). The assays were repeated in triplicate.

### Growth Curves

In order to investigate whether sub-MIC carnosol affects bacterial growth, growth curve analysis was performed. The growth curves of planktonic MRSA 4670 were described in a previous study (Shen et al., 2015). A total of 250 mL of MRSA 4670 (OD_600_ = 0.3) in TSB was distributed in five Erlenmeyer flasks. Carnosol at final concentrations of 0–256 μg/mL was added to the five Erlenmeyer flasks. The cultures were incubated at 37°C. The cell density was monitored at 600 nm spectrophotometrically every 0.5 h up to 6 h.

### Inhibition of Cell Attachment

A microplate biofilm assay was used to test the antiadhesion properties of carnosol on *S. aureus* according to A. Merghni et al. (2016) with minor modifications. Overnight cultures grown in TSB were diluted to 10^6^ cfu/mL in TSB. A 100 μL aliquot and 100 μL of sub-MIC (1/8 × to 1 × MIC) carnosol were transferred to a 96-well polystyrene microtiter plate. After incubation for 24 h at 37°C, the culture supernatant was discarded, and these test wells were washed with PBS to remove the loosely bound cells. The surface-attached cells in the test wells were stained with 200 μL of crystal violet (0.1%, w/v) at room temperature for 15 min. Dye taken up by the biofilm cells was dissolved in 95% (v/v) ethanol. The OD_570_ was measured using microplate reader.

### Sliding Motility Measurement of *S. aureus*

To investigate the sliding motility of *S. aureus* treated with carnosol, their spread ability on LB broth (30 g/L with soft agar (Qingdao Hope Bio-Technology Co., Ltd., Qingdao, Shandong, China) containing 2.4 g/L agar was tested in small petri dishes (Das et al., 2018). The spreading assay was carried out as described previously with some modifications (Kaito and Sekimizu, 2007a). Briefly, an aliquot of an overnight culture of MRSA 4670 (∼10^8^ cfu/mL) was administered in the center of the plates either treated or untreated with sub-MIC carnosol and dried at room temperature for 20 min. The plates were incubated for 48 h at 37°C. Colony growth expansions from the spot point were recorded.

### Reduction of Biofilm Growth and Development

The inhibition of biofilm formation by carnosol was indirectly assessed using the modified crystal violet assay (Alejo-Armijo et al., 2018). Overnight cultures grown in TSB were diluted to 10^5^ cfu/mL in TSB. A 200 μl aliquot was transferred to a 96-well microtiter plate for 24 h at 37°C, the medium was discarded, and then the wells were gently rinsed twice with PBS. A total of 200 μL of carnosol (1/2 × to 4 × MIC) was added to the wells at 37°C. The negative control was biofilm without carnosol. The positive control was biofilm with Licochalcone A (Mansite Biotechnology Co., Ltd., Chengdu, China). After 24 h, the plates were washed to remove non-adherent cells. Then, the wells were stained with 100 μL of 1% (w/v) crystal violet for 30 min at room temperature. In order to remove the unabsorbed stain, the plates were washed three times with PBS. The mean absorbance (OD570) of the crystal violet-stained biofilm cells was determined, and the percent inhibition of carnosol was determined by the following formula: [(OD growth control – OD sample)/OD growth control] × 100.

The metabolic activity of the biofilm formed was determined using the XTT (2,3-bis-(2-methoxy-4-nitro-5-sulfophenyl)-2H-tetrazolium-5-carboxanilalide) assay with minor modifications (Laird et al., 2012). Biofilms formed for 24 h in a 96-well microtiter plate. After treatment with carnosol (1/2 × to 4 × MIC) for 24 h, 20 μL of XTT in PBS (2 mg/mL)/phenazine methosulphate (PMS, 200 μmol/L) (Shanghai Sangon Biotech Co., Ltd., Shanghai, China) were added to the wells in the dark at 37°C for 4 h. The OD_450_ was measured using a microplate reader.

### Congo Red Binding Assay

The alteration in EPS production after treatment with carnosol was quantified by Congo red binding assay (Parai et al., 2020). *S. aureus* and sub-MIC concentrations of carnosol (1/8 × to 1/2 × MIC) were added to a 96-well polystyrene microtiter plate for 24 h at 37°C. The control was biofilm without carnosol. Following incubation, planktonic cells were discarded and adhered cells were washed with PBS. Persisting EPS were stained with 1% (w/v) Congo red for 30 min. Extra dye was dispensed off to resolubilize the bound dye with DMSO and the absorbance was recorded at 490 nm.

Percentage of Congo red binding = [A_*treated*_/A_*control*_] × 100

### eDNA Extraction

*Staphylococcus aureus* and sub-MIC concentrations of carnosol (1/8 × to 1/2 × MIC) were added to a 6-well polystyrene microtiter plate for 24 h at 37°C. The control was biofilm without carnosol. Following incubation, planktonic cells were carefully removed without disrupting the biofilm and 1 mL of TE buffer [pH = 8] (10 mM Tris and 1 mM EDTA) was added to biofilm. The biofilm matrix along with cells was completely scrapped out and transferred to 1.5 mL tubes. The tubes were centrifuged at 12, 000 rpm for 10 min to remove supernatant. The settled biofilm pellet was resuspended in 200 μL of TE buffer and vortexed for 1 h to disengage the biofilm components. The tubes were centrifuged to collect supernatant containing released eDNA (Valliammai et al., 2019). 10 μL of supernatant was run on 1% agarose gel and eDNA was visualized by ethidium bromide staining. Also, the extracted eDNA was quantified using NanoDrop spectrophotometer (Thermo Fisher Scientific).

### Confocal Laser Scanning Microscopy (CLSM) Analysis

The effect of carnosol on the disruption of preformed biofilms was observed by CLSM (Nikon, A1 PLUS, Tokyo, Japan) by generating biofilms on glass slides (2 × 2 cm) placed on 6-well microtiter plates. After treatment with carnosol at 37°C for 24 h, the glass slides were removed and washed twice with PBS to remove loosely adhered cells followed, by staining with a LIVE/DEAD BacLight bacterial viability kit (Molecular Probes, Eugene, OR, United States). Image analysis was performed using NIS-Elements.

### Metabolomic Analysis

Intracellular metabolites from five biological replicate were extracted using a cold methanol/chloroform/H_2_O extraction approach as previously reported (Stipetic et al., 2016). Briefly, The *S. aureus* 29213 planktonic cells was inoculated with an exponentially growing overnight culture at an initial optical density at 500 nm (OD500) of 0.06. At an OD of 0.5, bacterial cells were treated with 48 μg/mL (1.5 × MIC) (Dörries et al., 2014). Biofilms were grown for 24 h, then were treated with 128 μg/mL (4 × MIC) carnosol. The control groups were treated with the same volume of DMSO. After coincubation for 4 h, 10 mL of planktonic cells or biofilms from each biological sample was transferred to a 15 mL Eppendorf tube. The bacterial cells were harvested by centrifugation at 10000 *g* for 3 min, followed by PBS washes. The supernatants were removed, and the precipitate was rapidly frozen with liquid nitrogen to quench metabolism. Five milliliters of extraction solvent (methanol:chloroform:H_2_O 3:1:1 v:v:v) and 0.1 g of acid-washed glass beads (particle size, 0.1 mm) were added to each pellet, and the samples were vortexed vigorously for 1 min. The metabolites were extracted using ice bath ultrasound (400 W, 10 min) followed by centrifugation (12000 *g*, 5 min, 4°C). The supernatant was filtered through a 0.22 μm organic membrane filter and dried with a nitrogen blow and lyophilization. Lyophilized extracts were derivatized first with 50 μL of methoxyamine (20 mg/mL, J&K Scientific Ltd., Beijing, China) for 90 min at 30°C and then with 80 μL of N-methyl-N-(trimethylsilyl) trifluoroacetamide (J&K Scientific Ltd., Beijing, China) for 30 min at 37°C. After derivatization, the samples (1 μL) were injected in split mode (1:25) and analyzed by gas chromatography-mass spectrometry (6890N/5973, Agilent, United States). The metabolites were identified using full-scan monitoring with a detection slope of m/z 50-650, based on the National Institute of Standards and Technology (NIST) Mass Spectral Library with ChemStation software.

### Statistical Analysis

All experiments were performed in triplicate at a minimum. Each set of data was tested for normal distribution using skewness and kurtosis. The results were analyzed with one-way analysis of variance using SPSS 22. A value of *p* < 0.05 was considered statistically significant. Metabolomic analysis, including heat map, principal component analysis (PCA) analysis, metabolic pathways analysis, were performed using MetaboAnalyst version 4.0^1^ (Chong et al., 2018).

## Results

### Activity of Carnosol Against Planktonic *S. aureus*

The MICs and MIC breakpoints are shown in Table 1 according to Clinical and Laboratory Clinical and Laboratory Standards Institute [CLSI] (2012a, b). The target strains were sensitive to vancomycin and were resistant to penicillin G and gentamicin. However, most clinical strains were resistant to tetracyclines and oxacillin with the exception of MSSA 4611. Moreover, most clinical strains were resistant to levofloxacin and ciprofloxacin with the exception of MSSA 4611 and 4672. The MICs of carnosol for the target strains were 32 to 256 μg/mL.

**TABLE 1:** MICs of carnosol and 7 common antibiotics against 16 strains of *Staphylococcus aureus*.

**Strain**	**MIC (μg/mL)**							
	**CA**	**PEG**	**VAN**	**OXA**	**CIP**	**LVX**	**TET**	**GEN**
*S. aureus* 29123	32	0.05(S)	1(S)	< 0.125(S)	0.25(S)	< 0.25(S)	<0.125(S)	1(S)
MRSA 3183	64	32(R)	1(S)	> 256(R)	32(R)	16(R)	128(R)	16(R)
MRSA 2961	32	32(R)	1(S)	> 256(R)	32(R)	64(R)	16(R)	> 256(R)
MRSA 2047	64	16(R)	1(S)	64(R)	16(R)	16(R)	256(R)	256(R)
MRSA 41122	32	32(R)	1(S)	256(R)	128(R)	64(R)	8(I)	> 256(R)
MRSA 3335	32	32(R)	1(S)	> 256(R)	64(R)	64(R)	256(R)	256(R)
MRSA 4670	256	64(R)	1(S)	> 256(R)	64(R)	16(R)	128(R)	64(R)
MRSA 4642	256	256(R)	1(S)	> 256(R)	32(R)	32(R)	128(R)	128(R)
MSSA 4611	128	128(R)	1(S)	0.5(S)	0.5(S)	1(S)	0.5(S)	16(R)
MSSA 4672	32	8(R)	1(S)	8(R)	0.5(S)	0.5(S)	128(R)	8(R)
*S. aureus* 4643	128	128(R)	1(S)	> 256(R)	16(R)	32(R)	64(R)	32(R)
*S. aureus* 4644	128	128(R)	1(S)	> 256(R)	16(R)	32(R)	32(R)	32(R)
*S. aureus* 4563	128	> 256(R)	1(S)	> 256(R)	128(R)	128(R)	128(R)	> 256(R)
*S. aureus* 4612	128	> 256(R)	1(S)	> 256(R)	128(R)	64(R)	128(R)	32(R)
*S. aureus* 4623	128	> 256(R)	1(S)	256(R)	64(R)	32(R)	128(R)	8(R)
*S. aureus* 4474	32	128(R)	1(S)	4(R)	8(R)	2(I)	128(R)	16(R)

*PEG, Penicillin G; VAN, Vancomycin; OXA, Oxacillin; CIP, Ciprofloxacin; LVX, Levofloxacin; TET, Tetracyclines; GEN, Gentamicin; CA, Carnosol; S, sensitive; I, intermediate; R, resistant.*

**FIGURE 1:**
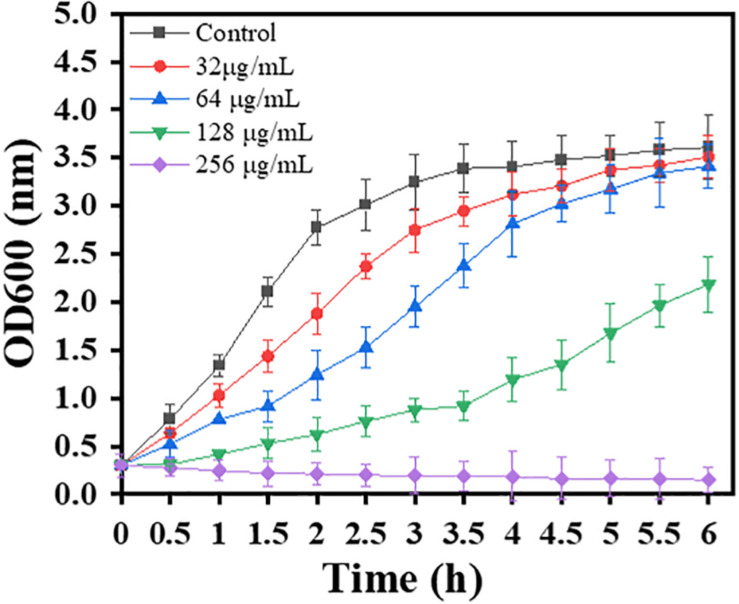
Growth curve for MRSA 4670 treated with carnosol.

Furthermore, the dynamics of the MRSA growth curve were monitored by measuring the OD600 of the control and bacterial solutions treated with different concentrations of carnosol. Time-dependent changes in bacterial growth were monitored at a regular interval of 0.5 h (up to 6 h) and are shown in Figure 1. The growth curve showed that at all tested concentrations, carnosol caused a significant growth delay of the bacterial cells; the slope of the bacterial growth curve continuously decreased with the increase in carnosol concentration over the first 4 h. Bacteria hardly grew for up to 6 h at 256 μg/mL (MIC concentration) of carnosol. These results showed that carnosol has antimicrobial activity against MRSA.

### Effects of Carnosol on Biofilm Formation

**FIGURE 2:**
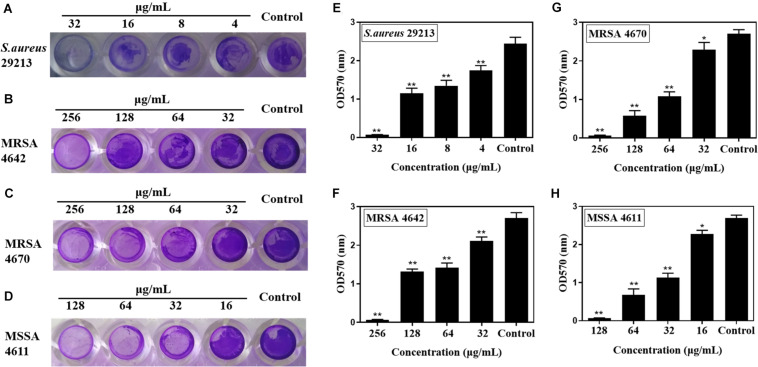
*In vitro* effects of sub-MIC concentrations of carnosol on *Staphylococcus aureus* biofilm adherence. The representative crystal violet-stained biomass of *S. aureus* 29213 **(A)**, MRSA 4642 **(B)**, MRSA 4670 **(C)** and MSSA 4611 **(D)** remaining on the microplate after carnosol treatment. Quantitative results of stained *S. aureus* 29213 **(E)**, MRSA 4642 **(F)**, MRSA 4670 **(G)** and MSSA 4611 **(H)** attached to the surface after washing in the presence of carnosol. Data are presented as mean ± SEM, *n* = 3, *p < 0.05, **p < 0.01, compared to the control group.

Subinhibitory concentrations of carnosol were tested in order to ascertain their effects on biofilm formation. There was a positive correlation between the concentration of the agents and the extent of inhibition of *S. aureus* biofilm formation (Figure 2). This shows a trend in the graphs in Figures 2A–D, with the most diluted agent having a biofilm formation close to that of the control (biofilm in saline). Sub-MIC concentrations of carnosol (1/8 × to 1 × MIC) exerted an anti-attachment effect on *S. aureus* (ATCC 29213, MRSA and MSSA) biofilms compared with the control (Figures 2E–H).

**FIGURE 3:**
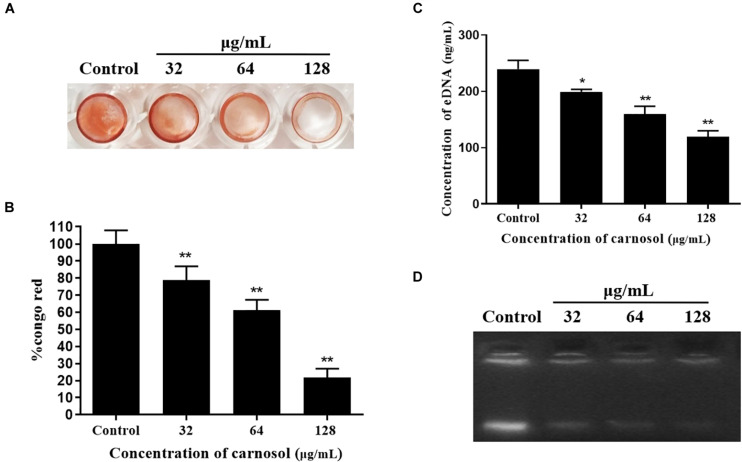
Carnosol treatment resulting in the reduction of eDNA and biofilm matrix in MRSA 4670 biofilm. **(A)** The representative congo red-stained biomass of MRSA 4670 remaining on the microplate after carnosol treatment. **(B)** Estimation of EPS matrix formation of the carnosol-treated biofilms by Congo red binding assay. **(C)** Estimation of eDNA of MRSA 4670 biofilm after carnosol treatment. **(D)** Corresponding representation of agarose gel electrophoresis of eDNA of MRSA 4670 biofilm after carnosol treatment. Data are presented as mean ± SEM, *n* = 3, *p < 0.05, **p < 0.01, compared to the control group.

Moreover, Carnosol diminished the EPS production of MRSA 4670 (Figure 3A). The percentage of EPS production was significantly reduced and a maximum 78.2% reduction was measured for 1/2 × MIC treated (Figure 3B). Concentration of eDNA of MRSA 4670 were given as bar graph indicates the reduction in eDNA with carnosol treatment (Figure 3C). Agarose gel electrophoresis exhibited a concentration dependent reduction in eDNA synthesis upon carnosol treatment compared with control (Figure 3D). These results showed that carnosol inhibited *Staphylococcal* biofilm formation.

### Bacterial Motility

**FIGURE 4:**
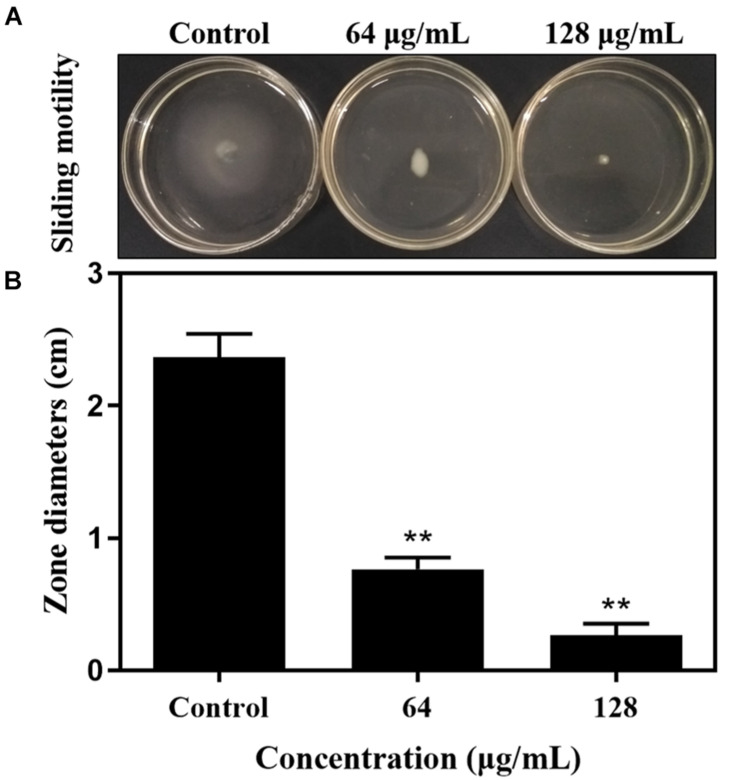
The sliding motility of MRSA 4670 was determined by measuring the extent of branching of microbial colony growth from point of inoculation **(A)**. The zone diameter was measured in centimeters and was expressed against carnosol **(B)**. Data are presented as mean ± SEM, *n* = 3, **p < 0.01, compared to the control group.

In order to explore the potential action mechanism of carnosol on pathogenic biofilms, we studied the effects of the drug on the sliding movement of *S. aureus*. The results in Figure 4A show that sub-MIC concentrations of carnosol attenuate the sliding of MRSA 4670 compared with the control. Moreover, the zone diameter remarkably decreased with 128 μg/mL carnosol treatment compared with the control group (Figure 4B). Thus, the inhibition of biofilm formation by carnosol is related to a reduction in bacterial motility.

### Disruption of Preformed Biofilms

**FIGURE 5:**
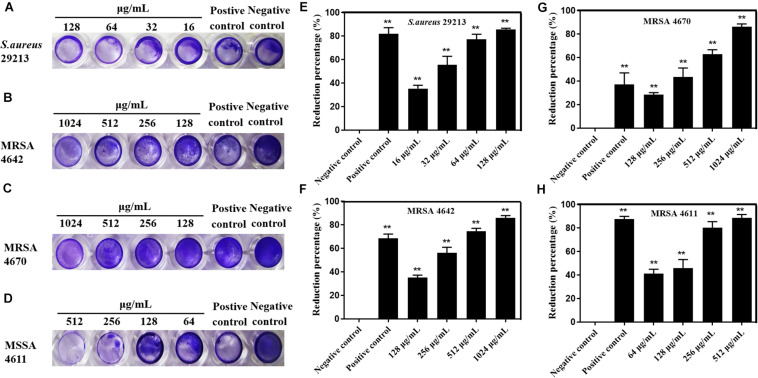
Effects of carnosol on the reduction of preformed biofilms of *Staphylococcus aureus* expressed as the reduction percent (%) evaluated by crystal violet staining. Images of preformed biofilm of *S. aureus* 29213 **(A)**, MRSA 4642 **(B)**, MRSA 4670 **(C)** and MSSA 4611 **(D)** in the presence of carnosol. Eradication percent values (%) of preformed biofilms of *S. aureus* 29213 **(E)**, MRSA 4642 **(F)**, MRSA 4670 **(G)** and MSSA 4611 **(H)** in the presence of carnosol. The positive control was biofilm with 16 μg/mL Licochalcone A (Shen et al., 2015). Data are presented as mean ± SEM, *n* = 3, *p < 0.05, **p < 0.01, compared to the control group.

**FIGURE 6:**
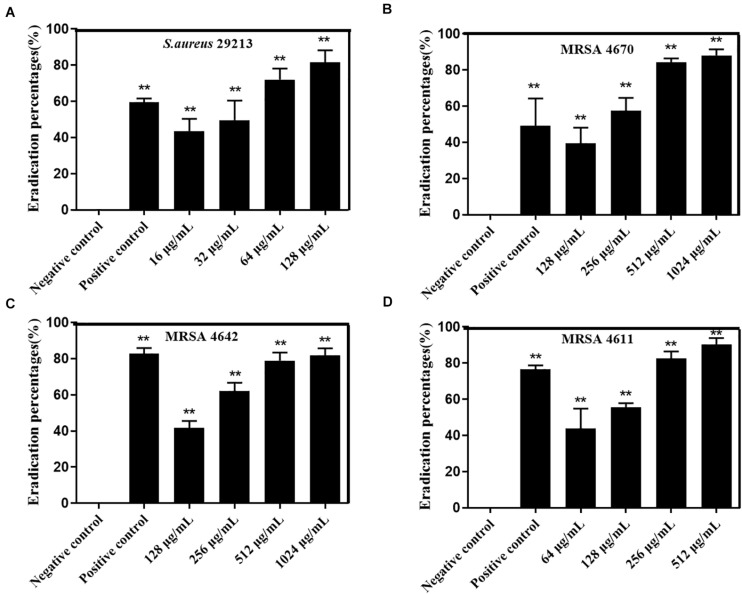
Effects of carnosol on the reduction of preformed biofilm of *Staphylococcus aureus* expressed as the eradication percent (%) evaluated by the XTT assay. Eradication percent values (%) of preformed biofilms of *S. aureus* 29213 **(A)**, MRSA 4642 **(B)**, MRSA 4670 **(C)** and MSSA 4611 **(D)** in the presence of carnosol. The positive control was biofilm with 16 μg/mL Licochalcone A (Shen et al., 2015). Data are presented as mean ± SEM, *n* = 3, *p < 0.05, **p < 0.01, compared to the control group.

In order to investigate the killing activity of carnosol against preformed *S. aureus* biofilms, the biofilm eradication activity of carnosol was measured by two methods (Figures 5, 6). Assessment of biofilm biomass by crystal violet staining showed that carnosol (1/2 × to 4 × MIC) had a killing effect on preformed biofilms (Figures 5A–D). Additionally, compared with the control group, carnosol significantly attenuated biofilm of ATCC 29213, MRSA 4642, MRSA 4670 and MSSA 4611, respectively (Figures 5E–H). These results showed that carnosol is able to eradicate preformed biofilms.

Moreover, assessment of biofilm metabolic activity by the XTT reduction assay showed that carnosol significantly reduced the metabolic activity of the biofilms produced by ATCC29213, MRSA4642, MRSA 4670 and MSSA 4611, respectively (Figures 6A–D). These results showed that carnosol directly killed *S. aureus* biofilms.

**FIGURE 7:**
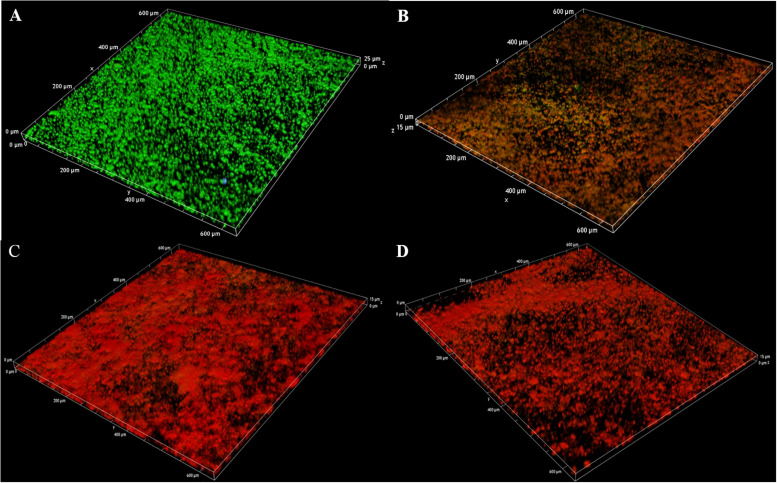
Confocal laser scanning microscopy 3D images of LIVE/DEAD-stained MRSA 4670 biofilms grown on coverslide disks: control **(A)**, MRSA 4670 treated with 128 μg/mL carnosol **(B)**, MRSA 4670 treated with 256 μg/mL carnosol **(C)** and MRSA 4670 treated with 512 μg/mL carnosol **(D)**.

In addition, the 3D image of preformed biofilms was observed by using CLSM with carnosol treatment at 37°C for 24 h. Red and green fluorescence represented dead cells and live cells, respectively. In the control experiment, green fluorescence labeled the microorganisms showed the formation of a very dense microbial biofilm network on the glass surface (Figure 7A). However, with carnosol treatment, Figures 7B–D shows that dead cells (red) showed the largest increases, live cells (green) had the greatest decreases in biofilm. This suggests that carnosol could penetrate the biofilm and disrupt the preformed biofilms.

### Carnosol Alters the *S. aureus* Metabolic Profile

**FIGURE 8:**
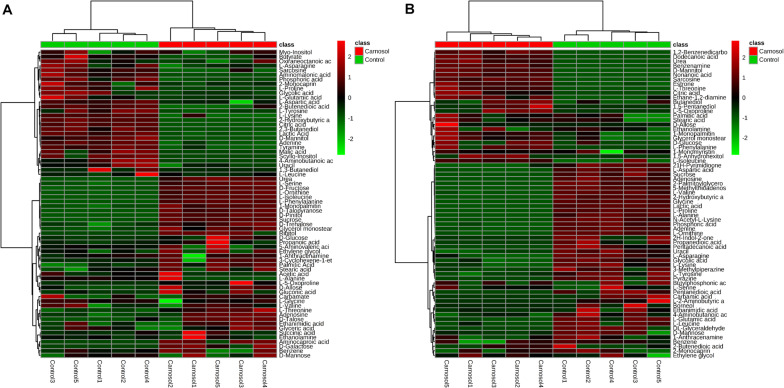
Heat map representation of the metabolites in the *Staphylococcus aureus* 29213 planktonic cells and biofilms that were exposed to the carnosol. Each column represents one biological replicate, and each row represents one detected metabolite. **(A)** The *S. aureus* 29213 planktonic cells were treated with 48 μg/mL (1.5 × MIC) carnosol (*n* = 5). The control groups were treated with the same volume of DMSO (*n* = 5). **(B)** The *S. aureus* 29213 biofilms were treated with 128 μg/mL (4 × MIC) carnosol (*n* = 5). The control groups were treated with the same volume of DMSO (*n* = 5). The up-regulated and down-regulated metabolites were shown in red and green, respectively.

**FIGURE 9:**
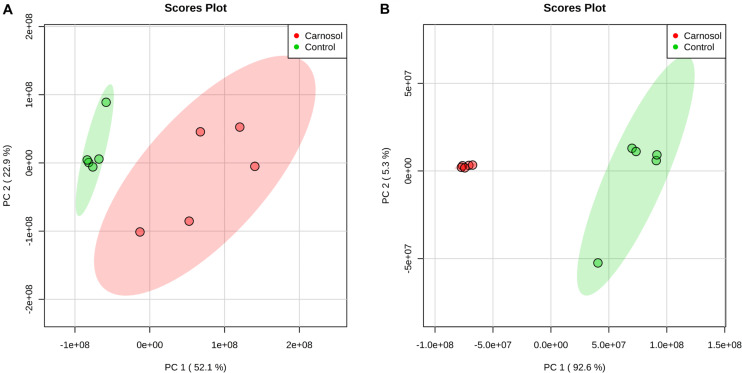
PCA score plot of metabolites in *Staphylococcus aureus* 29213 planktonic cells and biofilms treated with carnosol. **(A)** The *S. aureus* 29213 planktonic cells were treated with 48 μg/mL (1.5 × MIC) carnosol (*n* = 5). The control groups were treated with the same volume of DMSO (*n* = 5). **(B)** The *S. aureus* 29213 biofilms were treated with 128 μg/mL (4 × MIC) carnosol (*n* = 5). The control groups were treated with the same volume of DMSO (*n* = 5).

**FIGURE 10:**
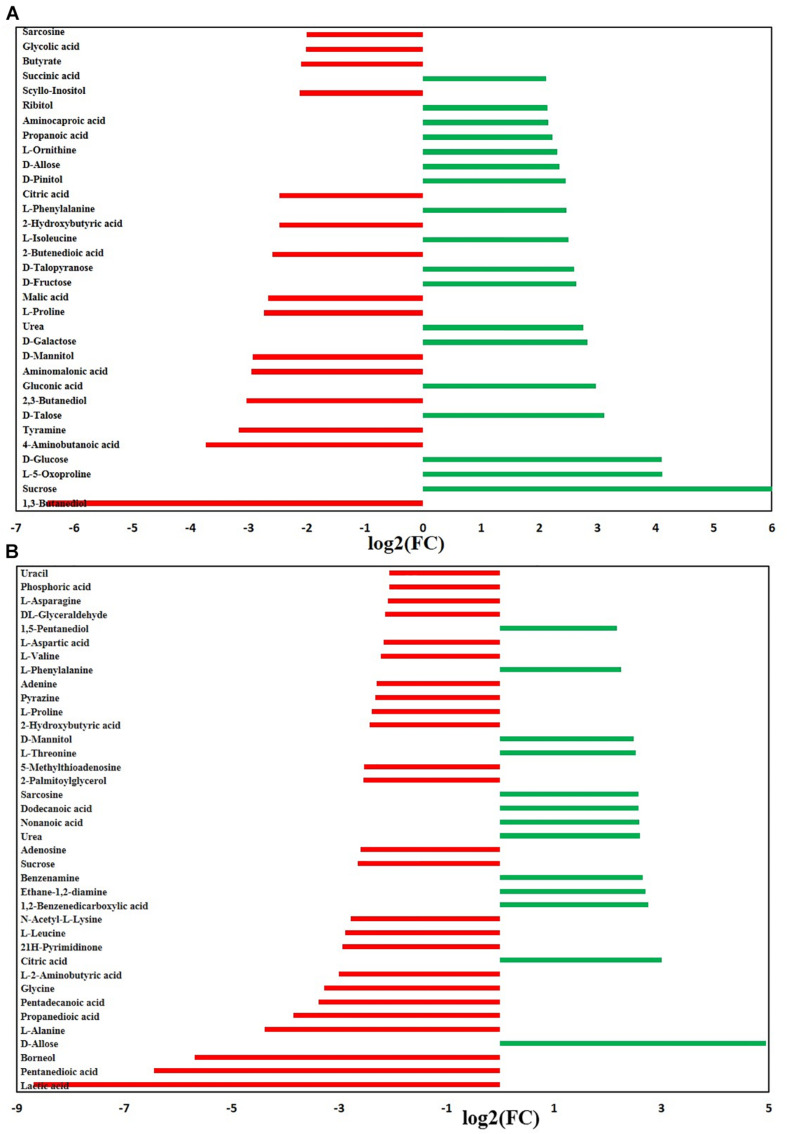
Significantly altered metabolites in the *Staphylococcus aureus* 29213 planktonic cells and biofilms that were exposed to the carnosol. The *S. aureus* 29213 planktonic **(A)** and biofilm **(B)** cells were treated with 48 and 128 μg/mL carnosol, respectively. The control groups were treated with the same volume of DMSO. (^∗∗^ ≥ 2.0-log_2_-fold, *p* ≤ 0.05).

**FIGURE 11:**
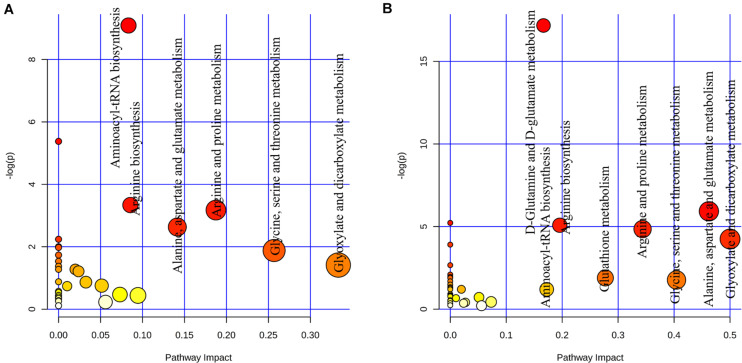
Analysis of metabolic pathways of *Staphylococcus aureus* 29213 planktonic cells and biofilms that were exposed to the carnosol. **(A)** The *S. aureus* 29213 planktonic cells were treated with 48 μg/mL (1.5 × MIC) carnosol (*n* = 5). The control groups were treated with the same volume of DMSO (*n* = 5). **(B)**The *S. aureus* 29213 biofilms were treated with 128 μg/mL (4 × MIC) carnosol (*n* = 5). The control groups were treated with the same volume of DMSO (*n* = 5). Each circle represents a metabolic pathway, with red indicating a higher impact and yellow indicating a lower impact. The pathways with impact values > 0.05 were determined to have significant changes. The size of the circles indicates the relative impact value.

We further investigated the metabolic profile of *S. aureus* ATCC 29213 under carnosol stress by non-targeted metabolomics. According to the MIC assays, 1.5 × MIC (48 μg/mL) and 4 × MIC (128 μg/mL) were used for planktonic cells and biofilms, respectively. The control groups were coincubated with the same volume of DMSO. The identified metabolites included amino acids, carbohydrates, fatty acids, small molecule acids and other biomolecules. The relative levels of the metabolites are presented using heat maps (Figure 8). In this heatmap, each column represents one biological replicate of the *S. aureus* sample, and each row represents one targeted metabolite. In contrast to the control group, hierarchical clustering (HCL) analysis (Figure 8) showed that carnosol induced obvious metabolite alterations in both planktonic cells and biofilms. To further directly evaluate the induced metabolic alterations and help establish whether differences in metabolic profiles exist among the groups, pattern recognition techniques such as principal component analysis (PCA) were applied to the data analysis. As shown in Figure 9, the two-dimensional score plots of the PCA models demonstrated that these groups were separated from each other and from the control group. The clear separation from the control group indicates that the metabolic perturbations were associated with carnosol treatment. In the planktonic cells, 18 and 15 metabolites were significantly up-regulated and down-regulated compared with the control group, respectively (Figure 10A). In the biofilms, 13 and 25 metabolites were significantly up-regulated and down-regulated compared with the control group, respectively (Figure 10B). Moreover, L-Ornithine and L-Glutamic acid were reduced by 1.9 and 1.7 times in *S. aureus* biofilms with carnosol treatment. Based on the KEGG pathway database, the significantly altered metabolic pathways were further analyzed. The significantly altered metabolic pathways upon carnosol treatment in *S. aureus* planktonic cells and biofilms were highly associated with the perturbation of glyoxylate and dicarboxylate metabolism, glycine, serine and threonine metabolism, arginine and proline metabolism, alanine, aspartate and glutamate metabolism, arginine biosynthesis, and aminoacyl-tRNA biosynthesis (Figure 11). In addition, glutathione metabolism, D-glutamine and D-glutamate metabolism were also significantly changed in biofilms (Figure 11B).

## Discussion

The prevalence of drug-resistant bacterial infections has become a major threat to human health. The clinical refractory nature of *S. aureus* is closely related to its ability to form biofilms, which exhibit greater drug resistance and often lead to long-term infection (Lîduma et al., 2012). Over the past two decades, the emergence of new antibiotics on the market has made little progress compared with the rapid rise of multidrug-resistant bacteria (Chopra et al., 2015). Plants and other natural materials, have been shown to be valuable sources of useful new antimicrobials and have been used since ancient times to attempt to cure for bacterial infections. In the present work, we investigated the antistaphylococcal activity and antibiofilm effects of carnosol.

In our study, fifteen clinical strains were found to be resistant to almost six conventional antibiotics, penicillin G, oxacillin, ciprofloxacin, levofloxacin, tetracyclines and gentamicin, with the exception of vancomycin. This result suggest that bacterial resistance is a very serious problem in the clinical treatment of *S. aureus*. The MICs of carnosol for *S. aureus* strains were 32 to 256 μg/mL (Table 1), indicating that carnosol has potential *in vitro* bactericidal activity against *S. aureus*. The effects of natural antibacterial agents, such as licochalcone A, gallidermin, PPAP 23 and rhodomyrtone, against *S. aureus* have been reported with MIC values ranging from 0.5–16 μg/mL (Saising et al., 2012, 2015; Götz et al., 2014; Shen et al., 2015; Wang et al., 2019). In contrast to these natural antibacterial agents, bactericidal concentration of carnosol is relatively high, which is an acceptable dose for human health. Carnosol is a constituent of rosemary, which has been approved as a food additive. In China and the European Union, depending on the food type, the addition of rosemary extract is permitted to a maximum concentration of ∼300.0–700.0 μg/mL and ∼30.0–250.0 μg/mL, respectively (Commission regulation (Eu) No 1129/2011 of 11 November 2011 amending, 2011 and National Health and Family Planning Commission [NHFPC], 2014). Carnosol with daily oral administration or intraperitoneally is well tolerated in several animal studies (Johnson, 2011). Previous research showed that dietary administration of 0.1% carnosol significantly decreased intestinal tumor multiplicity and wouldn’t change animal weight among treatment groups during the experiment for 10 weeks in C57BL/6J/Min/ + (Min/ +) mouse (Moran et al., 2005). The injection of camosol at 200 mg/kg for 5 days or diets supplemented 1% carnosol 14 days had no effect on animal weights in female Sprague-Dawley rats (Singletary, 1996). Oral administration of carnosol at 30 mg/kg five days weekly for 28 days to 22Rv1 PCa xenografted mice suppressed tumor growth (Johnson et al., 2010). Toxicological assays of oral rosemary extracts at dosages of 180–400 mg/kg/bw/day to male and female rats, which is equivalent to 20–60 mg/kg/day carnosol, lasted 13 weeks and showed no toxicity. The adult mean intake of this extract was estimated to be 500–1500 mg of carnosol per day (Del Baño et al., 2006).

Biofilms are networks of microbial communities. The characteristic is the production of polysaccharide intercellular adhesin, in which the cells are embedded and protected against adverse environmental condition (Heilmann et al., 1996; Mack et al., 1996; Götz, 2002). Moreover, this structure is more resistant to desiccation, grazing, and antimicrobial agents than planktonic cells (Kot et al., 2018; Suresh et al., 2019). It is widely recognized that the prevention of biofilm adhesion is a way to solve the problem of biofilms (Soto et al., 2006). In this study, our results showed that sub-MIC concentrations of carnosol exerted an anti-attachment effect in *S. aureus* (ATCC 29213, MRSA and MSSA) compared with the control (Figure 2). Exopolysaccharides (EPS) and eDNA are components of biofilms (Valliammai et al., 2019). Therefore, EPS and eDNA were detected. Carnosol treatment greatly reduced EPS and eDNA in MRSA 4670 biofilm (Figure 3). These results showed that carnosol effectively inhibits the formation of biofilm.

Motility, one of the most important features in microbial physiology, plays a key role in bacterial surface colonization and subsequent formation of biofilms (Das et al., 2016). Motility is divided into six types, i.e., swimming, swarming, gliding, twitching, sliding, and darting (Henrichsen, 1972). *S. aureus* is a non-flagellated gram-positive bacterium that spreads on surfaces by sliding mechanisms. This sliding motility is produced by the expansive (Henrichsen, 1972; Kaito and Sekimizu, 2007b). It has been known that the sliding motility of *S. aureus* enhances microbial colonization that can result in the formation of microbial biofilm over a surface (Das et al., 2016, 2018; Farha et al., 2020). According to previous research, subinhibitory concentration of drug was selected to investigate *S. aureus* sliding movement (Das et al., 2016, 2018; Farha et al., 2020). Our results showed that the attenuation effect of sub-MIC carnosol concentrations on MRSA 4670 slipping was dose-dependent (Figure 4). This suggests that the inhibitory effect of carnosol on *S. aureus* biofilms is related to reducing bacterial motility. Likewise, other reports shown that many natural compounds (e.g., terpenoid(+)-nootkatone, 3-Amino-4-aminoximidofurazan derivatives, coumarin derivatives) have capability to interfere with bacterial motility and limiting biofilm development of *S. aureus* (Das et al., 2016, 2018; Farha et al., 2020).

*In vitro*, biofilm assays can be performed on microtiter plate or glass surfaces (Cramton et al., 2001). In this study, two methods were used to evaluate the antibiofilm activity of carnosol on *S. aureus* in microtiter plate. The crystal violet and the XTT assay showed that carnosol (2 × MIC) exhibited kill preformed biofilms activity, with inhibition rate over 60% in test strain (Figures 5, 6). Moreover, MRSA 4670 biofilm formation was prevented by carnosol, which was further confirmed by CLSM, where the figures showed that the structure of the biofilm was markedly disorganized with 512 μg/mL (2 × MIC) carnosol treatment (Figure 7). These results showed that carnosol express low activity with already-established biofilms.

Over the last decades, transcriptomic and proteomic analysis have been used to identify genes and protein up- or down-regulated in bacterial biofilms (Resch et al., 2006). In contrast to genes and proteins, metabolites provide the most direct assessment of cellular phenotype, and metabolomics is the technique that is most suited to quantitative and dynamic monitoring of variations in bacterial metabolism in response to particular environmental conditions. By studying it directly, unique snapshots of underlying physiological processes can be obtained. Metabolomics has grown in recent years as a powerful tool both for complementing other omics studies and for investigating the immense metabolic diversity found in prokaryotes (Stipetic et al., 2016; Kiamco et al., 2018).

Post-treatment metabolic perturbation can play a major role in understanding the effectiveness of carnosol killing and inhibitory functions (Figures 8–11). Some altered metabolic pathways (glycine, serine and threonine metabolism, arginine and proline metabolism, alanine, aspartate and glutamate metabolism) have also been found in *S. aureus* under antibiotic stress, such as after treatment with ampicillin, kanamycin and norfloxacin (Schelli et al., 2017; Li and Zhu, 2018). This suggests that carnosol may have an antibacterial mechanism similar to these antibiotics. However, the changed metabolic pathway of arginine biosynthesis and aminoacyl-tRNA biosynthesis did not appear in *S. aureus* under the pressure of conventional antibiotics. We speculate that carnosol may also have a new antibacterial mechanism that is different from conventional antibiotics, which is of great significance in overcoming the resistance of *S. aureus*.

Glutamic acid, glutamine and arginine (and its derivatives) are the most representative amino acids, being at the same time potential nitrogen, carbon and energy sources (Rinaldo et al., 2018). Moreover, glutamate stimulates biofilm dispersion by producing matrix-degrading enzymes (Petrova and Sauer, 2016). A number of studies have linked resistance to osmolytes related to proline, glycine, and glutamate metabolism, which is affected the diffusivity of free molecules such as nutrients within the biofilm (Burg and Ferraris, 2008). After exposure to carnosol, intracellular levels of proline, glycine and glutamic acid markedly decreased in biofilm (Figure 10B). This suggests that *S. aureus* takes up proline, glycine and glutamic acid as osmoprotectants against carnosol stress.

Furthermore, previous studies suggest that arginine metabolism and catabolism play an important role in biofilm survival (Wu et al., 2010). In the study, pathway intermediates of arginine metabolism: glutamic acid, L-Ornithine, L-Proline and 4-Aminobutanoic acid were detected to be significantly down-regulated in the biofilm samples (Figure 10B), suggesting that they are expended in response to carnosol stress. Further research is needed to uncover and identify the potential new mechanisms.

In conclusion, carnosol show potential activity with planktonic cells and limit biofilm development is related to reducing bacterial motility, EPS and eDNA. But, carnosol express low activity with already-established biofilms. Moreover, carnosol also alter metabolic pathways of *S. aureus* planktonic cells and biofilms. The lead compound carnosol also can be modified to develop new compounds with better antibacterial activity, which will provide alternative strategies for overcoming the drug resistance of *S. aureus*.

## Data Availability Statement

All datasets generated for this study are included in the article/supplementary material.

## Author Contributions

FS did the data acquisition, data analysis, data interpretation, writing of the manuscript and revising of the manuscript. CG did the data acquisition and data analysis. PY did the data analysis, data interpretation and revising of the manuscript. All authors contributed to the article and approved the submitted version.

## Conflict of Interest

The authors declare that the research was conducted in the absence of any commercial or financial relationships that could be construed as a potential conflict of interest.

## Footnotes

1 http://www.metaboanalyst.ca
